# Supplementary material for: Self-amplifying RNA SARS-CoV-2 lipid nanoparticle vaccine candidate induces high neutralizing antibody titers in mice
Source: Nat Commun. 2020 Jul 9;11:3523. doi: 10.1038/s41467-020-17409-9 (PMC7347890; doi:10.1038/s41467-020-17409-9)
Supplement: Supplementary file 1 — Supplementary Information [file 41467_2020_17409_MOESM1_ESM.pdf]

Supplementary Information for:

Self-amplifying RNA SARS-CoV-2 lipid nanoparticle vaccine candidate induces high neutralizing antibody titers in mice

Paul F. McKay[1], Kai Hu[1], Anna K. Blakney[1], Karnyart Samnuan[1], Jonathan C. Brown[1], Rebecca Penn[1], Jie Zhou[1], Clément R. Bouton[1], Paul Rogers[1], Krunal Polra[1], Paulo J.C. Lin[2], Christopher Barbosa[2], Ying K. Tam[2], Wendy S. Barclay[1], Robin J. Shattock[1,*]

[1] Department of Infectious Diseases, Imperial College London, Norfolk Place, London, United Kingdom W2 1PG
[2]Acuitas Therapeutics, Vancouver, British Columbia, Canada, V6T 1Z3

*Corresponding author. Email: r.shattock@imperial.ac.uk

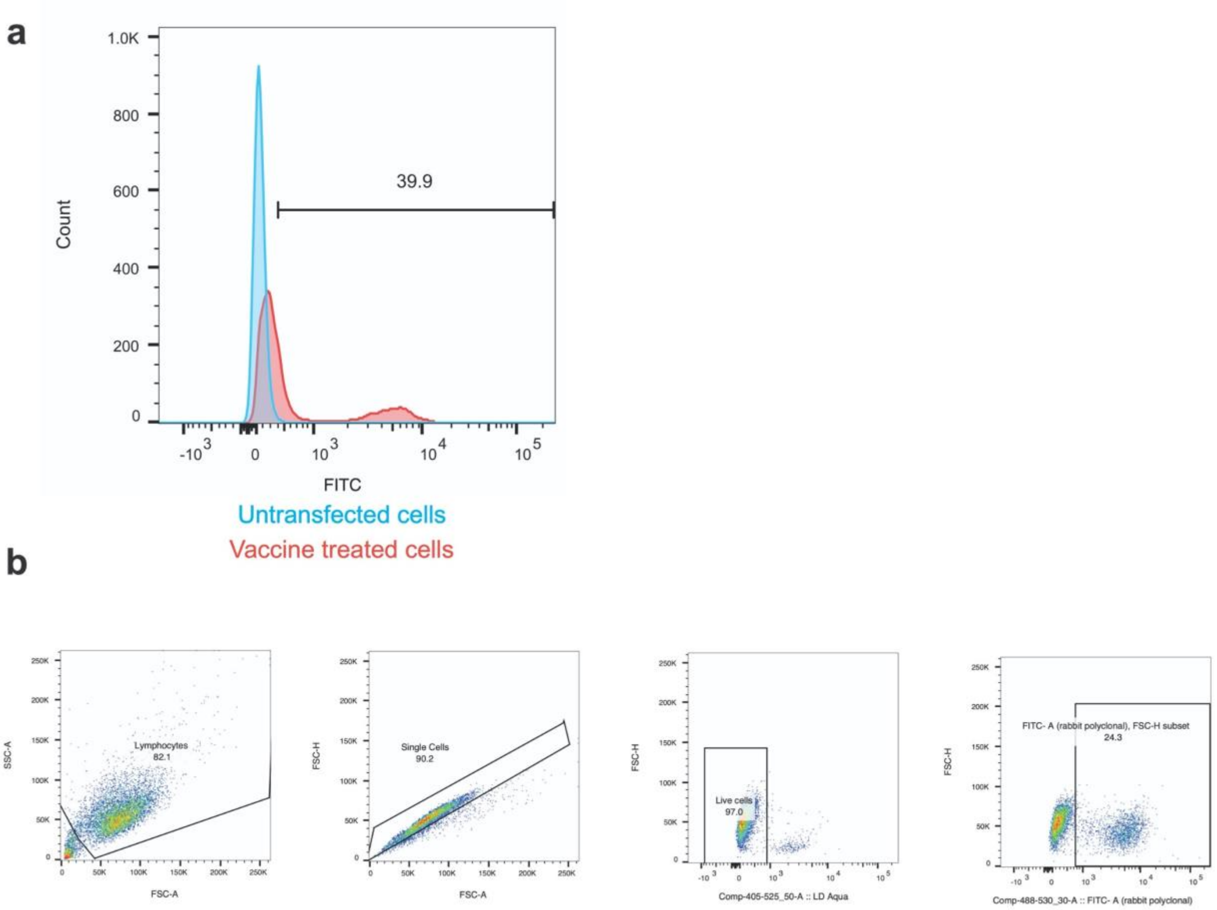

Supplementary Figure 1. Flow cytometry of HEK293T.17 cells transfected with saRNA expressing membrane bound SARS-CoV-2 spike protein. HEK293T.17 cells were transfected with 1 µg of saRNA encoding a membrane bound SARS-CoV-2 spike protein complexed with Lipofectamine MessengerMAX™ and stained with a fixable aqua live/dead stain, as well as SARS-CoV spike protein polyclonal antibody as a FITC-labelled secondary antibody.

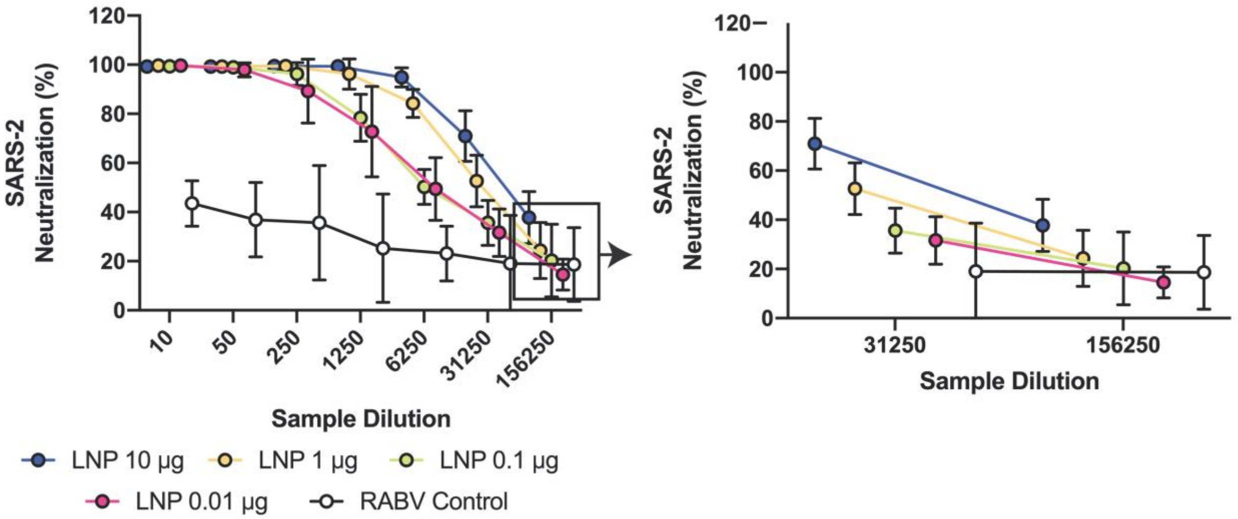

Supplementary Figure 2. Quantification of antibody-dependent enhancement (ADE) via wild type virus neutralization of sera from BALB/c mice vaccinated with doses of LNP-formulated saRNA ranging from 0.01-10 µg of saRNA with n=7 biologically independent animals. Line and error bars indicated mean ± SD.

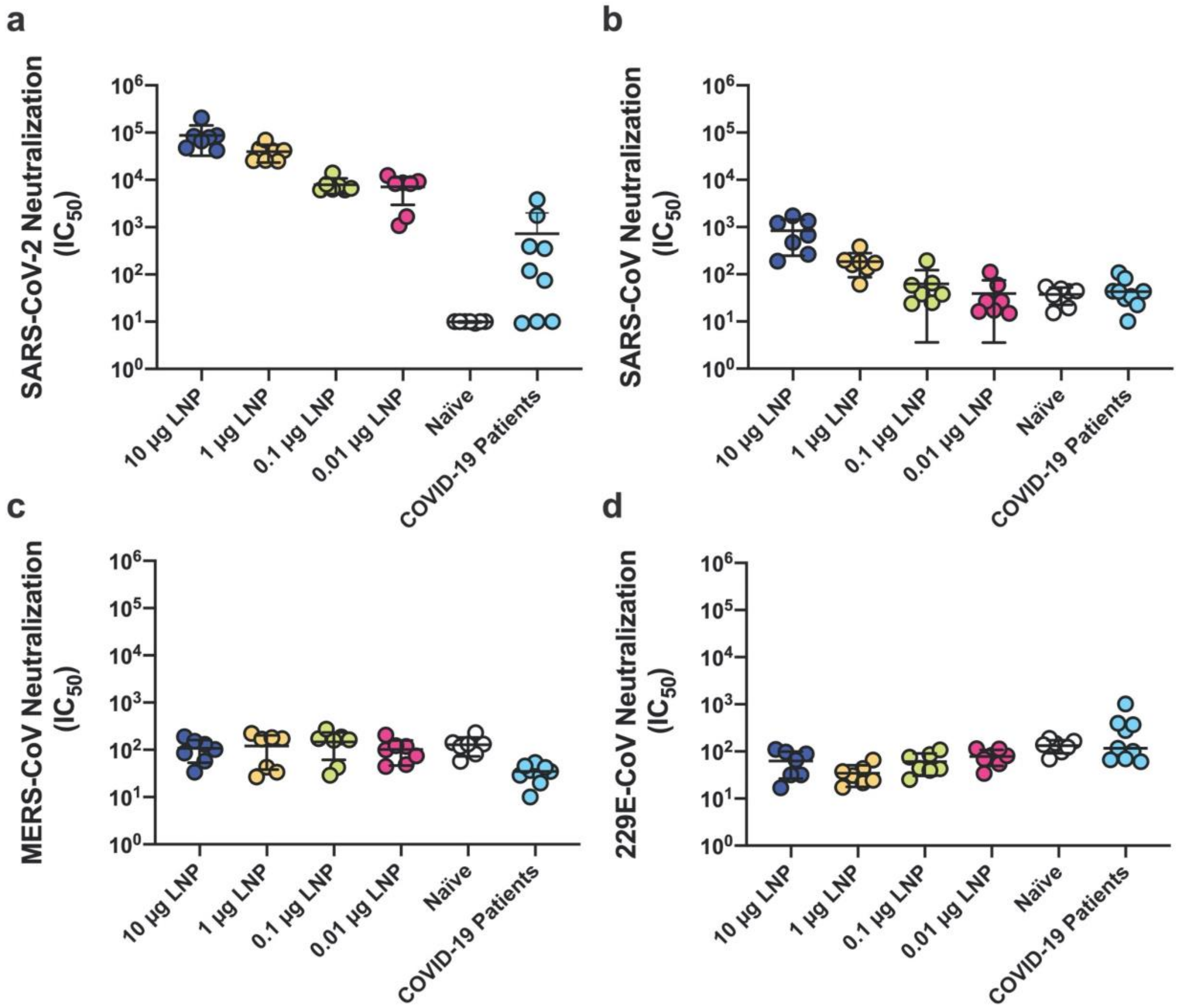

Supplementary Figure 3. Pseudotyped virus neutralization of sera from BALB/c mice vaccinated with LNP formulations (n=7 biologically independent animals) and human patients (n=9 biologically independent samples) after recovery from COVID-19. Line and error bars indicated mean ± SD.

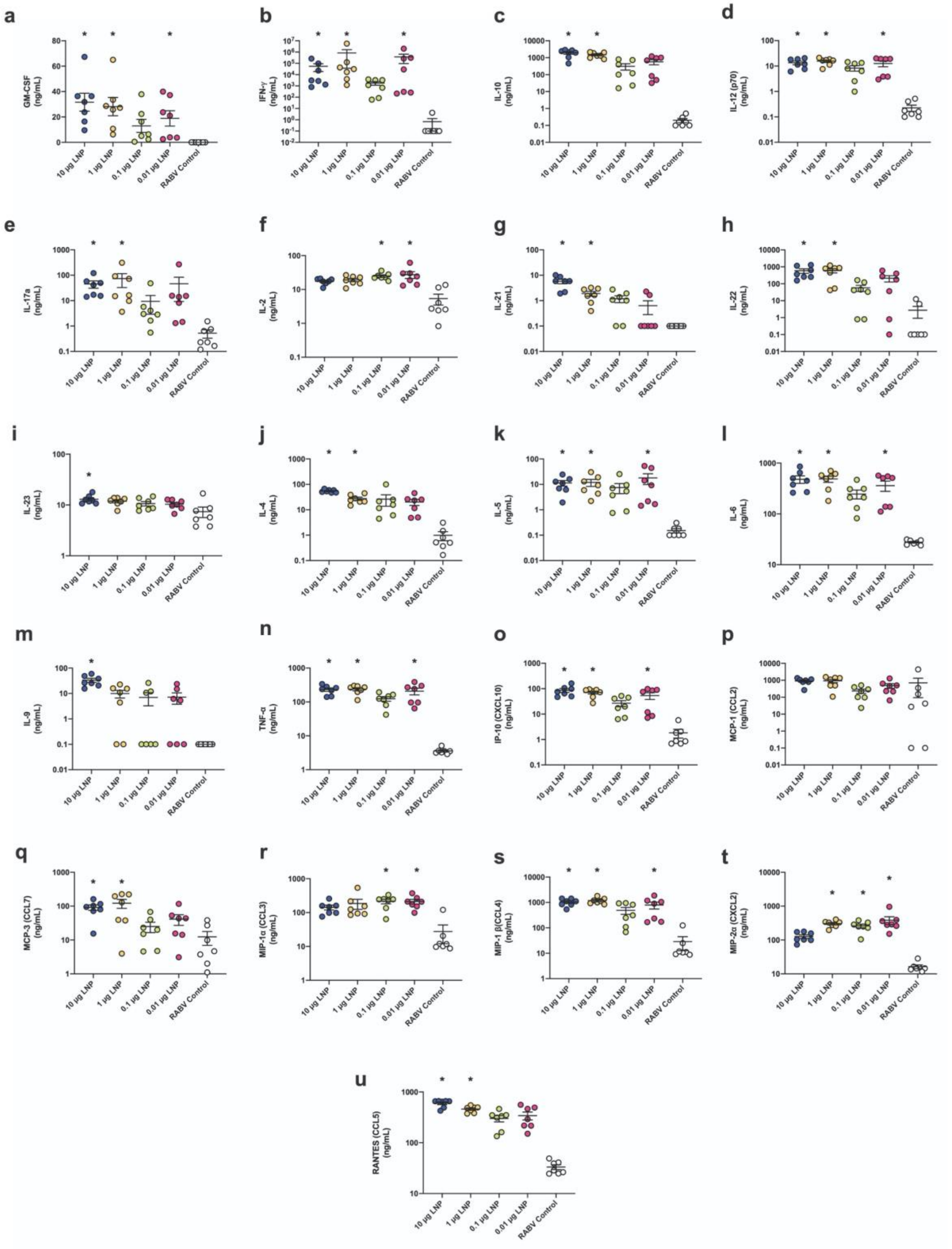

Supplementary Figure 4. Cytokine response of re-stimulated splenocytes of vaccinated mice (n=7 biologically independent animals) with recombinant SARS-CoV-2 protein. * indicates significance of p<0.05 as determined by a Kruskal-Wallis test. Line and error bars indicated mean ± SD.

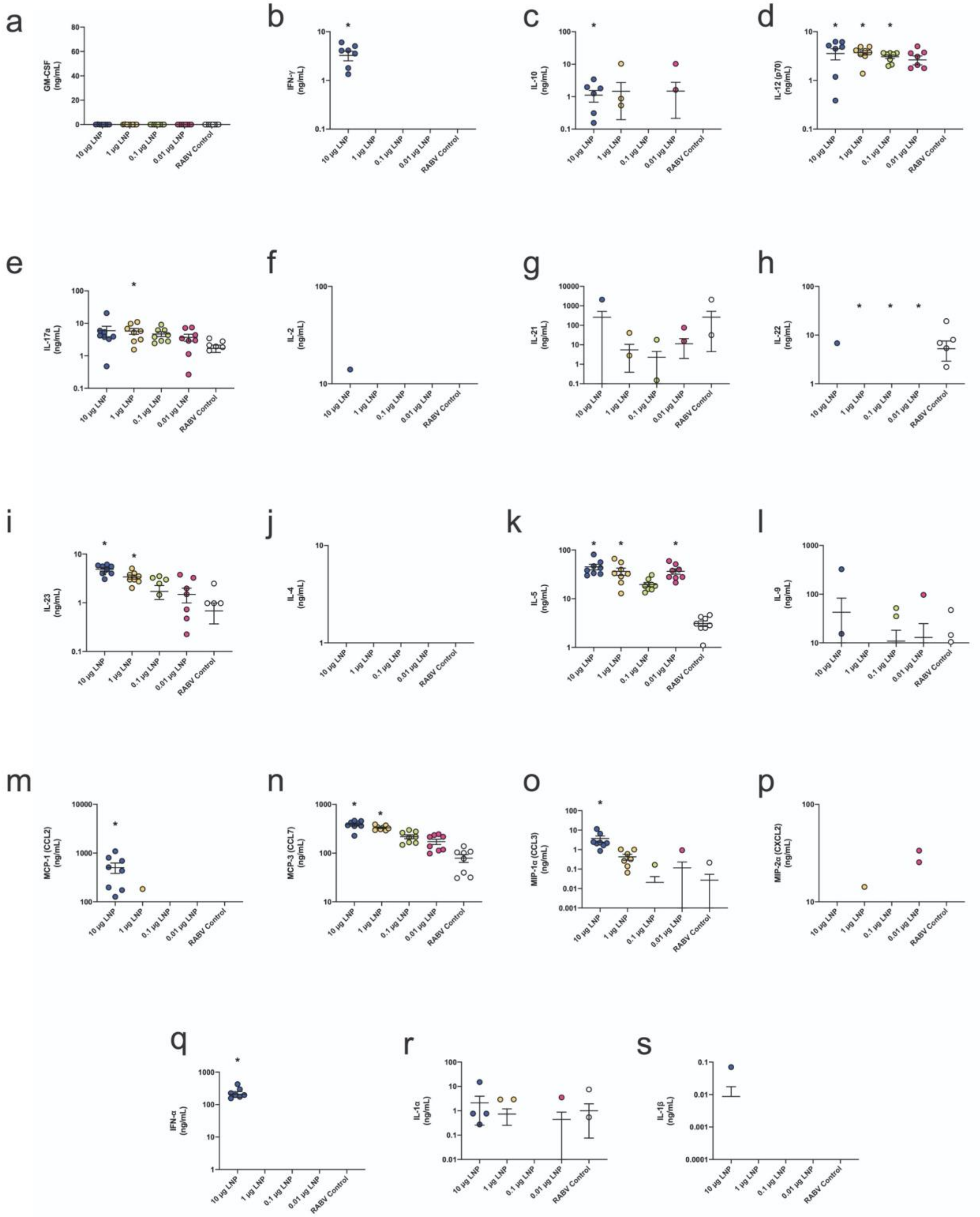

Supplementary Figure 5. Cytokine response in the sera of mice (n=7 biologically independent animals) 4 hours after vaccination with SARS-CoV-2 LNP vaccine. * indicates significance of p<0.05 as determined by a Kruskal-Wallis test. Line and error bars indicated mean ± SD.

AUGGGCGGCGCAUGAGAGAAGCCCAGACCAAUUACCUACCCAAAAUGGAGAAAGUUCAC
GUUGACAUCGAGGAAGACAGCCCAUUCCUCAGAGCUUUGCAGCGGAGCUUCCCGCAGU
UUGAGGUAGAAGCCAAGCAGGUCACUGAUAAUGACCAUGCUAAUGCCAGAGCGUUUUC
GCAUCUGGCUUCAAAACUGAUCGAAACGGAGGUGGACCCAUCCGACACGAUCCUUGACA
UUGGAAGUGCGCCCGCCCGCAGAAUGUAUUCUAAGCACAAGUAUCAUUGUAUCUGUCC
GAUGAGAUGUGCGGAAGAUCCGGACAGAUUGUAUAAGUAUGCAACUAAGCUGAAGAAAA
ACUGUAAGGAAAUAACUGAUAAGGAAUUGGACAAGAAAAUGAAGGAGCUGGCCGCCGUC
AUGAGCGACCCUGACCUGGAAACUGAGACUAUGUGCCUCCACGACGACGAGUCGUGUC
GCUACGAAGGGCAAGUCGCUGUUUACCAGGAUGUAUACGCGGUUGACGGACCGACAAG
UCUCUAUCACCAAGCCAAUAAGGGAGUUAGAGUCGCCUACUGGAUAGGCUUUGACACCA
CCCCUUUUAUGUUUAAGAACUUGGCUGGAGCAUAUCCAUCAUACUCUACCAACUGGGCC
GACGAAACCGUGUUAACGGCUCGUAACAUAGGCCUAUGCAGCUCUGACGUUAUGGAGC
GGUCACGUAGAGGGAUGUCCAUUCUUAGAAAGAAGUAUUUGAAACCAUCCAACAAUGUU
CUAUUCUCUGUUGGCUCGACCAUCUACCACGAGAAGAGGGACUUACUGAGGAGCUGGC
ACCUGCCGUCUGUAUUUCACUUACGUGGCAAGCAAAAUUACACAUGUCGGUGUGAGACU
AUAGUUAGUUGCGACGGGUACGUCGUUAAAAGAAUAGCUAUCAGUCCAGGCCUGUAUG
GGAAGCCUUCAGGCUAUGCUGCUACGAUGCACCGCGAGGGAUUCUUGUGCUGCAAAGU
GACAGACACAUUGAACGGGGAGAGGGUCUCUUUUCCCGUGUGCACGUAUGUGCCAGCU
ACAUUGUGUGACCAAAUGACUGGCAUACUGGCAACAGAUGUCAGUGCGGACGACGCGC
AAAAACUGCUGGUUGGGCUCAACCAGCGUAUAGUCGUCAACGGUCGCACCCAGAGAAAC
ACCAAUACCAUGAAAAAUUACCUUUUGCCCGUAGUGGCCCAGGCAUUUGCUAGGUGGG
CAAAGGAAUAUAAGGAAGAUCAAGAAGAUGAAAGGCCACUAGGACUACGAGAUAGACAG
UUAGUCAUGGGGUGUUGUUGGGCUUUUAGAAGGCACAAGAUAACAUCUAUUUAUAAGC
GCCCGGAUACCCAAACCAUCAUCAAAGUGAACAGCGAUUUCCACUCAUUCGUGCUGCCC
AGGAUAGGCAGUAACACAUUGGAGAUCGGGCUGAGAACAAGAAUCAGGAAAAUGUUAGA
GGAGCACAAGGAGCCGUCACCUCUCAUUACCGCCGAGGACGUACAAGAAGCUAAGUGC
GCAGCCGAUGAGGCUAAGGAGGUGCGUGAAGCCGAGGAGUUGCGCGCAGCUCUACCAC
CUUUGGCAGCUGAUGUUGAGGAGCCCACUCUGGAAGCCGAUGUCGACUUGAUGUUACA
AGAGGCUGGGGCCGGCUCAGUGGAGACACCUCGUGGCUUGAUAAAGGUUACCAGCUAC
GAUGGCGAGGACAAGAUCGGCUCUUACGCUGUGCUUUCUCCGCAGGCUGUACUCAAGA
GUGAAAAAUUAUCUUGCAUCCACCCUCUCGCUGAACAAGUCAUAGUGAUAACACACUCU
GGCCGAAAAGGGCGUUAUGCCGUGGAACCAUACCAUGGUAAAGUAGUGGUGCCAGAGG
GACAUGCAAUACCCGUCCAGGACUUUCAAGCUCUGAGUGAAAGUGCCACCAUUGUGUAC
AACGAACGUGAGUUCGUAAACAGGUACCUGCACCAUAUUGCCACACAUGGAGGAGCGCU
GAACACUGAUGAAGAAUAUUACAAAACUGUCAAGCCCAGCGAGCACGACGGCGAAUACC
UGUACGACAUCGACAGGAAACAGUGCGUCAAGAAAGAACUAGUCACUGGGCUAGGGCUC
ACAGGCGAGCUGGUGGAUCCUCCCUUCCAUGAAUUCGCCUACGAGAGUCUGAGAACAC
GACCAGCCGCUCCUUACCAAGUACCAACCAUAGGGGUGUAUGGCGUGCCAGGAUCAGG
CAAGUCUGGCAUCAUUAAAAGCGCAGUCACCAAAAAAGAUCUAGUGGUGAGCGCCAAGA
AAGAAAACUGUGCAGAAAUUAUAAGGGACGUCAAGAAAAUGAAAGGGCUGGACGUCAAU
GCCAGAACUGUGGACUCAGUGCUCUUGAAUGGAUGCAAACACCCCGUAGAGACCCUGU
AUAUUGACGAAGCUUUUGCUUGUCAUGCAGGUACUCUCAGAGCGCUCAUAGCCAUUAUA
AGACCUAAAAAGGCAGUGCUCUGCGGGGAUCCCAAACAGUGCGGUUUUUUAACAUGAU
GUGCCUGAAAGUGCAUUUUAACCACGAGAUUUGCACACAAGUCUUCCACAAAAGCAUCU
CUCGCCGUUGCACUAAAUCUGUGACUUCGGUCGUCUCAACCUUGUUUUACGACAAAAAA
AUGAGAACGACGAAUCCGAAAGAGACUAAGAUUGUGAUUGACACUACCGGCAGUACCAA
ACCUAAGCAGGACGAUCUCAUUCUCACUUGUUUCAGAGGGUGGGUGAAGCAGUUGCAA
AUAGAUUACAAAGGCAACGAAAUAAUGACGGCAGCUGCCUCUCAAGGGCUGACCCGUAA
AGGUGUGUAUGCCGUUCGGUACAAGGUGAAUGAAAAUCCUCUGUACGCACCCACCUCA
GAACAUGUGAACGUCCUACUGACCCGCACGGAGGACCGCAUCGUGUGGAAAACACUAG
CCGGCGACCCAUGGAUAAAAACACUGACUGCCAAGUACCCUGGGAAUUUCACUGCCACG

AUAGAGGAGUGGCAAGCAGAGCAUGAUGCCAUCAUGAGGCACAUCUUGGAGAGACCGG
ACCCUACCGACGUCUUCCAGAAUAAGGCAAACGUGUGUUGGGCCAAGGCUUUAGUGCC
GGUGCUGAAGACCGCUGGCAUAGACAUGACCACUGAACAAUGGAACACUGUGGAUUAUU
UUGAAACGGACAAAGCUCACUCAGCAGAGAUAGUAUUGAACCAACUAUGCGUGAGGUUC
UUUGGACUCGAUCUGGACUCCGGUCUAUUUUCUGCACCCACUGUUCCGUUAUCCAUUA
GGAAUAAUCACUGGGAUAACUCCCCGUCGCCUAACAUGUACGGGCUGAAUAAAGAAGUG
GUCCGUCAGCUCUCUCGCAGGUACCCACAACUGCCUCGGGCAGUUGCCACUGGAAGAG
UCUAUGACAUGAACACUGGUACACUGCGCAAUUAUGAUCCGCGCAUAAACCUAGUACCU
GUAAACAGAAGACUGCCUCAUGCUUUAGUCCUCCACCAUAAUGAACACCCACAGAGUGA
CUUUUCUUCAUUCGUCAGCAAAUUGAAGGGCAGAACUGUCCUGGUGGUCGGGGAAAAG
UUGUCCGUCCCAGGCAAAAUGGUUGACUGGUUGUCAGACCGGCCUGAGGCUACCUUCA
GAGCUCGGCUGGAUUUAGGCAUCCCAGGUGAUGUGCCCAAAUAUGACAUAAUAUUUGU
UAAUGUGAGGACCCCAUAUAAAUACCAUCACUAUCAGCAGUGUGAAGACCAUGCCAUUA
AGCUUAGCAUGUUGACCAAGAAAGCUUGUCUGCAUCUGAAUCCCGGCGGAACCUGUGU
CAGCAUAGGUUAUGGUUACGCUGACAGGGCCAGCGAAAGCAUCAUUGGUGCUAUAGCG
CGGCAGUUCAAGUUUUCCCGGGUAUGCAAACCGAAAUCCUCACUUGAAGAGACGGAAGU
UCUGUUUGUAUUCAUUGGGUACGAUCGCAAGGCCCGUACGCACAAUUCUUACAAGCUU
UCAUCAACCUUGACCAACAUUUAUACAGGUUCCAGACUCCACGAAGCCGGAUGUGCACC
CUCAUAUCAUGUGGUGCGAGGGGAUAUUGCCACGGCCACCGAAGGAGUGAUUAUAAAU
GCUGCUAACAGCAAAGGACAACCUGGCGGAGGGGUGUGCGGAGCGCUGUAUAAGAAAU
UCCCGGAAAGCUUCGAUUUACAGCCGAUCGAAGUAGGAAAAGCGCGACUGGUCAAAGG
UGCAGCUAAACAUAUCAUUCAUGCCGUAGGACCAAACUUCAACAAAGUUUCGGAGGUUG
AAGGUGACAAACAGUUGGCAGAGGCUUAUGAGUCCAUCGCUAAGAUUGUCAACGAUAAC
AAUUACAAGUCAGUAGCGAUUCCACUGUUGUCCACCGGCAUCUUUUCCGGGAACAAAGA
UCGACUAACCCAAUCAUUGAACCAUUUGCUGACAGCUUUAGACACCACUGAUGCAGAUG
UAGCCAUAUACUGCAGGGACAAGAAAUGGGAAAUGACUCUCAAGGAAGCAGUGGCUAGG
AGAGAAGCAGUGGAGGAGAUAUGCAUAUCCGACGACUCUUCAGUGACAGAACCUGAUGC
AGAGCUGGUGAGGGUGCAUCCGAAGAGUUCUUUGGCUGGAAGGAAGGGCUACAGCACA
AGCGAUGGCAAAACUUUCUCAUAUUUGGAAGGGACCAAGUUUCACCAGGCGGCCAAGGA
UAUAGCAGAAAUUAAUGCCAUGUGGCCCGUUGCAACGGAGGCCAAUGAGCAGGUAUGC
AUGUAUAUCCUCGGAGAAAGCAUGAGCAGUAUUAGGUCGAAAUGCCCCGUCGAAGAGUC
GGAAGCCUCCACACCACCUAGCACGCUGCCUUGCUUGUGCAUCCAUGCCAUGACUCCA
GAAAGAGUACAGCGCCUAAAAGCCUCACGUCCAGAACAAAUUACUGUGUGCUCAUCCUU
UCCAUUGCCGAAGUAUAGAAUCACUGGUGUGCAGAAGAUCCAAUGCUCCCAGCCUAUAU
UGUUCUCACCGAAAGUGCCUGCGUAUAUUCAUCCAAGGAAGUAUCUCGUGGAAACACCA
CCGGUAGACGAGACUCCGGAGCCAUCGGCAGAGAACCAAUCCACAGAGGGGACACCUG
AACAACCACCACUUAUAACCGAGGAUGAGACCAGGACUAGAACGCCUGAGCCGAUCAUC
AUCGAAGAGGAAGAAGAGGAUAGCAUAAGUUUGCUGUCAGAUGGCCCGACCCACCAGG
UGCUGCAAGUCGAGGCAGACAUUCACGGGCCGCCCUCUGUAUCUAGCUCAUCCUGGUC
CAUUCCUCAUGCAUCCGACUUUGAUGUGGACAGUUUAUCCAUACUUGACACCCUGGAG
GGAGCUAGCGUGACCAGCGGGGCAACGUCAGCCGAGACUAACUCUUACUUCGCAAAGA
GUAUGGAGUUUCUGGCGCGACCGGUGCCUGCGCCUCGAACAGUAUUCAGGAACCCUCC
ACAUCCCGCUCCGCGCACAAGAACACCGUCACUUGCACCCAGCAGGGCCUGCUCGAGAA
CCAGCCUAGUUUCCACCCCGCCAGGCGUGAAUAGGGUGAUCACUAGAGAGGAGCUCGA
GGCGCUUACCCCGUCACGCACUCCUAGCAGGUCGGUCUCGAGAACCAGCCUGGUCUCC
AACCCGCCAGGCGUAAAUAGGGUGAUUACAAGAGAGGAGUUUGAGGCGUUCGUAGCAC
AACAACAAUGACGGUUUGAUGCGGGUGCAUACAUCUUUUCCUCCGACACCGGUCAAGG
GCAUUUACAACAAAAAUCAGUAAGGCAAACGGUGCUAUCCGAAGUGGUGUUGGAGAGGA
CCGAAUUGGAGAUUUCGUAUGCCCCGCGCCUCGACCAAGAAAAAGAAGAAUUACUACGC
AAGAAAUUACAGUUAAAUCCCACACCUGCUAACAGAAGCAGAUACCAGUCCAGGAAGGU
GGAGAACAUGAAAGCCAUAACAGCUAGACGUAUUCUGCAAGGCCUAGGGCAUUAUUUGA

AGGCAGAAGGAAAAGUGGAGUGCUACCGAACCCUGCAUCCUGUUCCUUUGUAUUCAUC
UAGUGUGAACCGUGCCUUUUCAAGCCCCAAGGUCGCAGUGGAAGCCUGUAACGCCAUG
UUGAAAGAGAACUUUCCGACUGUGGCUUCUUACUGUAUUAUUCCAGAGUACGAUGCCUA
UUUGGACAUGGUUGACGGAGCUUCAUGCUGCUUAGACACUGCCAGUUUUUGCCCUGCA
AAGCUGCGCAGCUUUCCAAAGAAACACUCCUAUUUGGAACCCACAAUACGAUCGGCAGU
GCCUUCAGCGAUCCAGAACACGCUCCAGAACGUCCUGGCAGCUGCCACAAAAAGAAAUU
GCAAUGUCACGCAAAUGAGAGAAUUGCCCGUAUUGGAUUCGGCGGCCUUUAAUGUGGA
AUGCUUCAAGAAAUAUGCGUGUAAUAAUGAAUAUUGGGAAACGUUUAAAGAAAACCCCA
UCAGGCUUACUGAAGAAAACGUGGUAAAUUACAUUACCAAAUUAAAAGGACCAAAAGCUG
CUGCUCUUUUUGCGAAGACACAUAAUUUGAAUAUGUUGCAGGACAUACCAAUGGACAGG
UUUGUAAUGGACUUAAAGAGAGACGUGAAAGUGACUCCAGGAACAAAACAUACUGAAGA
ACGGCCCAAGGUACAGGUGAUCCAGGCUGCCGAUCCGCUAGCAACAGCGUAUCUGUGC
GGAAUCCACCGAGAGCUGGUUAGGAGAUUAAAUGCGGUCCUGCUUCCGAACAUUCAUA
CACUGUUUGAUAUGUCGGCUGAAGACUUUGACGCUAUUAUAGCCGAGCACUUCCAGCC
UGGGGAUUGUGUUCUGGAAACUGACAUCGCGUCGUUUGAUAAAAGUGAGGACGACGCC
AUGGCUCUGACCGCGUUAAUGAUUCUGGAAGACUUAGGUGUGGACGCAGAGCUGUUGA
CGCUGAUUGAGGCGGCUUUCGGCGAAAUUUCAUCAAUACAUUUGCCCACUAAAACUAAA
UUUAAAUUCGGAGCCAUGAUGAAAUCUGGAAUGUUCCUCACACUGUUUGUGAACACAGU
CAUUAACAUUGUAAUCGCAAGCAGAGUGUUGAGAGAACGGCUAACCGGAUCACCAUGUG
CAGCAUUCAUUGGAGAUGACAAUAUCGUGAAAGGAGUCAAAUCGGACAAAUUAAUGGCA
GACAGGUGCGCCACCUGGUUGAAUAUGGAAGUCAAGAUUAUAGAUGCUGUGGUGGGCG
AGAAAGCGCCUUAUUUCUGUGGAGGGUUUAUUUUGUGUGACUCCGUGACCGGCACAGC
GUGCCGUGUGGCAGACCCCCUAAAAAGGCUGUUUAAGCUUGGCAAACCUCUGGCAGCA
GACGAUGAACAUGAUGAUGACAGGAGAAGGGCAUUGCAUGAAGAGUCAACACGCUGGAA
CCGAGUGGGUAUUCUUUCAGAGCUGUGCAAGGCAGUAGAAUCAAGGUAUGAAACCGUA
GGAACUUCCAUCAUAGUUAUGGCCAUGACUACUCUAGCUAGCAGUGUUAAAUCAUUCAG
CUACCUGAGAGGGGCCCCUAUAACUCUCUACGGCUAACCUGAAUGGACUACGACAUAGU
CUAGUCCGCCAAGUCUAGCAUAUGGCCACCAUGUUCGUGUUUCUGGUGCUGCUGCCUC
UGGUGUCCAGCCAGUGUGUGAACCUGACCACCAGAACACAGCUGCCUCCAGCCUACAC
CAACAGCUUUACCAGAGGCGUGUACUACCCCGACAAGGUGUUCAGAUCCAGCGUGCUG
CACUCUACCCAGGACCUGUUCCUGCCUUUCUUCAGCAACGUGACCUGGUUCCACGCCA
UCCACGUGUCCGGCACCAAUGGCACCAAGAGAUUCGACAACCCCGUGCUGCCCUUCAAC
GACGGGGUGUACUUUGCCAGCACCGAGAAGUCCAACAUCAUCAGAGGCUGGAUCUUCG
GCACCACACUGGACAGCAAGACCCAGAGCCUGCUGAUCGUGAACAACGCCACCAACGUG
GUCAUCAAAGUGUGCGAGUUCCAGUUCUGCAACGACCCCUUCCUGGGCGUCUACUACC
ACAAGAACAACAAGAGCUGGAUGGAAAGCGAGUUCCGGGUGUACAGCAGCGCCAACAAC
UGCACCUUCGAGUACGUGUCCCAGCCUUUCCUGAUGGACCUGGAAGGCAAGCAGGGCA
ACUUCAAGAACCUGCGCGAGUUCGUGUUCAAGAACAUCGACGGCUACUUCAAGAUCUAC
AGCAAGCACACCCCUAUCAACCUCGUGCGGGAUCUGCCUCAGGGCUUCUCUGCUCUGG
AACCCCUGGUGGAUCUGCCCAUCGGCAUCAACAUCACCCGGUUUCAGACACUGCUGGC
CCUGCACAGAAGCUACCUGACACCUGGCGAUAGCAGCAGCGGAUGGACAGCUGGUGCC
GCCGCUUACUAUGUGGGCUACCUGCAGCCUAGAACCUUCCUGCUGAAGUACAACGAGA
ACGGCACCAUCACCGACGCCGUGGAUUGUGCCCUUGAUCCUCUGAGCGAGACAAAGUG
CACCCUGAAGUCCUUCACCGUGGAAAAGGGCAUCUACCAGACCAGCAACUUCCGGGUG
CAGCCCACCGAAUCCAUCGUGCGGUUCCCCAAUAUCACCAAUCUGUGCCCCUUCGGCG
AGGUGUUCAAUGCCACCAGAUUCGCCUCUGUGUACGCCUGGAACCGGAAGCGGAUCAG
CAAUUGCGUGGCCGACUACUCCGUGCUGUACAACUCCGCCAGCUUCAGCACCUUCAAG
UGCUACGGCGUGUCCCCUACCAAGCUGAACGACCUGUGCUUCACAAACGUGUACGCCG
ACAGCUUCGUGAUCCGGGGAGAUGAAGUGCGGCAGAUUGCCCCUGGACAGACAGGCAA
GAUCGCCGACUACAACUACAAGCUGCCCGACGACUUCACCGGCUGUGUGAUUGCCUGG
AACAGCAACAACCUGGACUCCAAAGUCGGCGGCAACUACAAUUACCUGUACCGGCUGUU

CCGGAAGUCCAAUCUGAAGCCCUUCGAGCGGGACAUCUCCACCGAGAUCUAUCAGGCC
GGCAGCACCCCUUGUAACGGCGUGGAAGGCUUCAACUGCUACUUCCCACUGCAGUCCU
ACGGCUUUCAGCCCACAAAUGGCGUGGGCUAUCAGCCCUACAGAGUGGUGGUGCUGAG
CUUCGAACUGCUGCAUGCCCCUGCCACAGUGUGCGGCCCUAAGAAAGCACCAAUCUC
GUGAAGAACAAAUGCGUGAACUUCAACUUCAACGGCCUGACCGGCACAGGCGUGCUGA
CAGAGAGCAACAAGAAGUUCCUGCCAUUCCAGCAGUUUGGCCGGGAUAUCGCCGAUAC
CACAGACGCCGUUAGAGAUCCCCAGACACUGGAAAUCCUGGACAUCACCCCUUGCAGCU
UCGGCGGAGUGUCUGUGAUCACCCCUGGCACCAACACCAGCAAUCAGGUGGCAGUGCU
GUACCAGGACGUGAACUGUACCGAAGUGCCCGUGGCCAUUCACGCCGAUCAGCUGACA
CCUACAUGGCGGGUGUACUCCACCGGCAGCAAUGUGUUUCAGACCAGAGCCGGCUGUC
UGAUCGGAGCCGAGCACGUGAACAAUAGCUACGAGUGCGACAUCCCCAUCGGCGCUGG
CAUCUGUGCCAGCUACCAGACACAGACAAACAGCCCCAGACGGGCCAGAUCUGUGGCCA
GCCAGAGCAUCAUUGCCUACACAAUGUCUCUGGGCGCCGAGAACAGCGUGGCCUACUC
CAACAACUCUAUCGCUAUCCCCACCAACUUCACCAUCAGCGUGACCACAGAGAUCCUGC
CUGUGUCCAUGACCAAGACCAGCGUGGACUGCACCAUGUACAUCUGCGGCGAUUCCAC
CGAGUGCUCCAACCUGCUGCUGCAGUACGGCAGCUUCUGCACCCAGCUGAAUAGAGCC
CUGACAGGGAUCGCCGUGGAACAGGACAAGAACACCCAAGAGGUGUUCGCCCAAGUGA
AGCAGAUCUACAAGACCCCUCCUAUCAAGGACUUCGGCGGCUUCAAUUUCAGCCAGAUU
CUGCCCGAUCCUAGCAAGCCCAGCAAGCGGAGCUUCAUCGAGGACCUGCUGUUCAACA
AAGUGACACUGGCCGACGCCGGCUUCAUCAAGCAGUAUGGCGAUUGUCUGGGCGACAU
UGCCGCCAGGGAUCUGAUUUGCGCCCAGAAGUUUAACGGACUGACAGUGCUGCCUCCU
CUGCUGACCGAUGAGAUGAUCGCCCAGUACACAUCUGCCCUGCUGGCCGGCACAAUCA
CAAGCGGCUGGACAUUUGGAGCUGGCGCCGCUCUGCAGAUCCCCUUUGCUAUGCAGAU
GGCCUACAGAUUCAACGGCAUCGGAGUGACCCAGAAUGUGCUGUACGAGAACCAGAAG
CUGAUCGCCAACCAGUUCAACAGCGCCAUCGGCAAGAUCCAGGACAGCCUGAGCAGCAC
AGCAAGCGCCCUGGGAAAGCUGCAGGACGUGGUCAACCAGAAUGCCCAGGCACUGAAC
ACCCUGGUCAAGCAGCUGUCCUCCAACUUCGGCGCCAUCAGCUCUGUGCUGAACGAUA
UCCUGAGCAGACUGGACCCUCCUGAGGCCGAGGUGCAGAUCGACAGACUGAUCACAGG
CAGACUGCAGAGCCUCCAGACAUACGUGACCCAGCAGCUGAUCAGAGCCGCCGAGAUUA
GAGCCUCUGCCAAUCUGGCCGCCACCAAGAUGUCUGAGUGUGUGCUGGGCCAGAGCAA
GAGAGUGGACUUUUGCGGCAAGGGCUACCACCUGAUGAGCUUCCCUCAGUCUGCCCCU
CACGGCGUGGUGUUUCUGCACGUGACAUACGUUCCCGCUCAAGAGAAGAAUUUCACCA
CCGCUCCAGCCAUCUGCCACGACGGCAAAGCCCACUUUCCUAGAGAAGGCGUGUUCGU
GUCCAACGGCACCCAUUGGUUCGUGACACAGCGGAACUUCUACGAGCCCCAGAUCAUCA
CCACCGACAACACCUUCGUGUCUGGCAACUGCGACGUCGUGAUCGGCAUUGUGAACAA
UACCGUGUACGACCCUCUGCAGCCCGAGCUGGACAGCUUCAAAGAGGAACUGGACAAG
UACUUUAAGAACCACACAAGCCCCGACGUGGACCUGGGCGAUAUCAGCGGAAUCAAUGC
CAGCGUCGUGAACAUCCAGAAAGAGAUCGACCGGCUGAACGAGGUGGCCAAGAAUCUG
AACGAGAGCCUGAUCGACCUGCAAGAACUGGGGAAGUACGAGCAGUACAUCAAGUGGC
CCUGGUACAUCUGGCUGGGCUUUAUCGCCGGACUGAUUGCCAUCGUGAUGGUCACAAU
CAUGCUGUGUUGCAUGACCAGCUGCUGUAGCUGCCUGAAGGGCUGUUGUAGCUGUGG
CAGCUGCUGCAAGUUCGACGAGGACGAUUCUGAGCCCGUGCUGAAGGGCGUGAAACUG
CACUACACAUGAGCGGCCGCGAAUUGGCAAGCUGCUUACAUAGAACUCGCGGCGAUUG
GCAUGCCGCCUUAAAAUUUUUAUUUUAUUUUUCUUUUCUUUUCCGAAUCGGAUUUUGUU
UUUAAUAUUUCAAAAAAAAAAAAAAAAAAAAAAAAAAAAAAAAAAAA

Supplementary Figure 6. Sequence of saRNA encoding NSP1-4 of VEEV and the spike glycoprotein of SARS-CoV-2.

Supplementary Table 1. Primers used for sequencing of SARS-CoV-2 saRNA vector.

**Sequencing Primer Name**	**Sequence**
nCoV FOR_1	AGAGCCTGCTGATCGTGAAC
nCoV FOR_2	TACCTGACACCTGGCGATAG
nCoV FOR_3	TGTCCCCTACCAAGCTGAAC
nCoV FOR_4	GAACTGCTGCATGCCCCT
nCoV FOR_5	CGCCCAAGTGAAGCAGAT
nCoV FOR_6	CGAGAACCAGAAGCTGATCG
nCoV FOR_7	ATGAGCTTCCCTCAGTCTGC
nCoV FOR_8	AAGAGATCGACCGGCTGAA
nCoV REV_1	GTGGTAGTAGACGCCCAGGA
nCoV REV_2	TCAGCAGGAAGGTTCTAGGC
nCoV REV_3	CAATCTGCCGCACTTCATCT
nCoV REV_4	CAGGCCGTTGAAGTTGAAGT
nCoV REV_5	GGCTGTTTGTCTGTGTCTGG
nCoV REV_6	TGCTAGGATCGGGCAGAAT
nCoV REV_7	GCGCTTGCTGTGCTGCTC
nCoV REV_8	GCGGTGGTGAAATTCTTCTC
nCoV REV_9	GGGCCACTTGATGTACTGCT
VEEV sgp FW	ATCATTCAGCTACCTGAGAGG
VEEVrep-Not1-Rev	CTATGTAAGCAGCTTGCCAATTC
