# Supplementary material for: Self-amplifying RNA SARS-CoV-2 lipid nanoparticle vaccine candidate induces high neutralizing antibody titers in mice
Source: Nat Commun. 2020 Jul 9;11:3523. doi: 10.1038/s41467-020-17409-9 (PMC7347890; doi:10.1038/s41467-020-17409-9)
Supplement: Supplementary file 3 — Reporting Summary [file 41467_2020_17409_MOESM3_ESM.pdf]

nature research

Corresponding author(s): Robin Shattock

Last updated by author(s): Jun 18, 2020

Reporting Summary

Nature Research wishes to improve the reproducibility of the work that we publish. This form provides structure for consistency and transparency in reporting. For further information on Nature Research policies, see Authors & Referees and the Editorial Policy Checklist .

Statistics

For all statistical analyses, confirm that the following items are present in the figure legend, table legend, main text, or Methods section.

n/a	Confirmed	
☐	☒	The exact sample size (n) for each experimental group/condition, given as a discrete number and unit of measurement
☐	☒	A statement on whether measurements were taken from distinct samples or whether the same sample was measured repeatedly
☐	☒	The statistical test(s) used AND whether they are one- or two-sided<br>*Only common tests should be described solely by name; describe more complex techniques in the Methods section.*
☐	☒	A description of all covariates tested
☐	☒	A description of any assumptions or corrections, such as tests of normality and adjustment for multiple comparisons
☐	☒	A full description of the statistical parameters including central tendency (e.g. means) or other basic estimates (e.g. regression coefficient) AND variation (e.g. standard deviation) or associated estimates of uncertainty (e.g. confidence intervals)
☐	☒	For null hypothesis testing, the test statistic (e.g. F, t, r) with confidence intervals, effect sizes, degrees of freedom and P value noted<br>*Give P values as exact values whenever suitable.*
☒	☐	For Bayesian analysis, information on the choice of priors and Markov chain Monte Carlo settings
☒	☐	For hierarchical and complex designs, identification of the appropriate level for tests and full reporting of outcomes
☒	☐	Estimates of effect sizes (e.g. Cohen's d, Pearson's r), indicating how they were calculated

Our web collection on statistics for biologists contains articles on many of the points above.

Software and code

Policy information about availability of computer code

Data collection	N/A
Data analysis	N/A

For manuscripts utilizing custom algorithms or software that are central to the research but not yet described in published literature, software must be made available to editors/reviewers. We strongly encourage code deposition in a community repository (e.g. GitHub). See the Nature Research guidelines for submitting code & software for further information.

Data

Policy information about availability of data

All manuscripts must include a data availability statement. This statement should provide the following information, where applicable:

- Accession codes, unique identifiers, or web links for publicly available datasets
- A list of figures that have associated raw data
- A description of any restrictions on data availability

The data that support the findings of this study are available from the corresponding author upon reasonable request.

Field-specific reporting

Please select the one below that is the best fit for your research. If you are not sure, read the appropriate sections before making your selection.

☒ Life sciences ☐ Behavioural & social sciences ☐ Ecological, evolutionary & environmental sciences

For a reference copy of the document with all sections, see nature.com/documents/nr-reporting-summary-flat.pdf

Life sciences study design

All studies must disclose on these points even when the disclosure is negative.

Sample size

We have extensively discussed the particular minimum group sizes necessary to provide adequate power in these immunogenicity experiments with professional statisticians and statistical services groups. A parametric Levene's test will be used to assess the assumption of homogeneity of the group variances at the 5% significance level. Datasets with at least three groups will be compared using an overall one-way ANOVA F-test or Kruskal-Wallis test (if parametric assumptions are not met) at the 5% significance level. Pairwise comparisons will be conducted using a two-sided Dunnett's or Dunn's test, respectively, if the overall test is significant. Datasets with two groups will be compared using a two-sided t-test or Wilcoxon Rank-Sum test, respectively. All significant pairwise comparisons will be reported at the 5% significance levels. The individual experiments carried out under this project licence are designed such that they do not require an unnecessary number of animals to accomplish experimental objectives. The total number of animals used in our studies was considered to be the minimum required to properly characterise the effects of the test item and provide >80% power to detect an effect size of 1 (between active and control) with 5% significance.

Therefore a power of 0.8 was used to calculate experimental group size. 0.8 was chosen on grounds of reduction and refinement. It is the power that gives the best ratio of likelihood of success in the context of the smallest group size. Whilst 0.75 would reduce group size, the likelihood of false negatives is greater and may lead to needless repetition of studies. Our main focus is on antibody titre, our pilot studies indicate that an increase of absorbance (d) of 0.5 was statistically significant, and that the standard deviation (s) was 0.25.

Using the calculation: $n = 1 + 2C(s/d)2$

to determine the necessary group size, where C is the constant (defined by the values of the probability ($\alpha = 0.05$) and the power ($1-\beta = 0.8$) = 7.85) and n = the group size required to fulfil the power requirement of the experiment.

This equation therefore requires a group size of n = 5 to fulfil the power requirement for our typical experiment.

Experimental bias by the researcher will be avoided as there will be no pre-selection of individual animals based on any inherent characteristic – eg weight, appearance etc. The animals will be adequately randomised prior to the start of the study by the animal technician randomly placing animals in cage groupings. Each cage will therefore receive the various vaccine formulations in a randomised manner.

Data exclusions

No data were excluded from the analyses.

Replication

Reproducibility of in vitro, ex vivo and in vivo experiments was verified where possible by repeating the experiment on a different day with a unique batch of materials.

Randomization

Samples were randomly allocated into groups by putting them into the appropriate groups sizes and then assigned a generic group number used in the blinding process.

Blinding

Investigators were blinded to sample groups during data collection and analysis by using generic group numbers.

Reporting for specific materials, systems and methods

We require information from authors about some types of materials, experimental systems and methods used in many studies. Here, indicate whether each material, system or method listed is relevant to your study. If you are not sure if a list item applies to your research, read the appropriate section before selecting a response.

Materials & experimental systems

n/a	Involved in the study	
☐	☒	Antibodies
☐	☒	Eukaryotic cell lines
☒	☐	Palaeontology
☐	☒	Animals and other organisms
☐	☒	Human research participants
☒	☐	Clinical data

Methods

n/a	Involved in the study	
☒	☐	ChIP-seq
☐	☒	Flow cytometry
☒	☐	MRI-based neuroimaging

Antibodies

Antibodies used

SARS-CoV Spike Protein Polyclonal Antibody (#PA1-41165) (Thermo Fisher Scientific)
FITC goat anti-rabbit IgG (#554020) (BD Pharmigen)
Anti-mouse Kappa (#1050-01) and Lambda (#1060-01) light chains (Southern Biotech)
Standard IgG (or IgG1 or IgG2) (Sigma)
Anti-mouse IgG-HRP (#1030-05), anti-mouse IgG1-HRP (#1070-05) or anti-mouse IgG2a-HRP (#1080-05) (Southern Biotech)

Validation	Validation for each antibody was confirmed by the manufacturer (Thermo Fisher, Sigma, BD Pharmigen or Southern Biotech) documentation is available on their website.

Eukaryotic cell lines

Policy information about cell lines

Cell line source(s)	HEK293T.17 cells were purchased from ATCC
Authentication	Cell lines were authenticated by supplier
Mycoplasma contamination	All cell lines routinely tested negative for mycoplasma contamination
Commonly misidentified lines (See ICLAC register)	N/A

Animals and other organisms

Policy information about studies involving animals; ARRIVE guidelines recommended for reporting animal research

Laboratory animals	BALB/c mice were used in these studies
Wild animals	N/A
Field-collected samples	N/A
Ethics oversight	All animals were handled in accordance with the UK Home Office Animals Scientific Procedures Act 1986 and with an internal ethics board and UK government approved project (P63FE629C) and personal license (IF15DBDA3)

Note that full information on the approval of the study protocol must also be provided in the manuscript.

Human research participants

Policy information about studies involving human research participants

Population characteristics	The samples taken from the human research participants were all from people who had been hospitalised with confirmed SARS-CoV-2 infection and had recovered from infrection, this being confirmed by PCR. Their samples were donated to the Communicable Diseases Research Tissue Bank, Section of Virology, Imperial College London, following written informed consent. The samples consist of serum collected from venous blood draws and the processing and storage is not expected to have altered the characteristics of the sample.
Recruitment	Participants were recruited by the tissue bank team based on their confirmed infection with COVID-19 and recovery based on PCR analysis.
Ethics oversight	The Communicable Diseases Research Tissue Bank, Section of Virology, Imperial College London is approved by the National Research Ethics Service, South Central Committee Oxford C (Ref 15/SC/0089).

Note that full information on the approval of the study protocol must also be provided in the manuscript.

Flow Cytometry

Plots

Confirm that:

- [x] The axis labels state the marker and fluorochrome used (e.g. CD4-FITC).
- [x] The axis scales are clearly visible. Include numbers along axes only for bottom left plot of group (a 'group' is an analysis of identical markers).
- [x] All plots are contour plots with outliers or pseudocolor plots.
- [x] A numerical value for number of cells or percentage (with statistics) is provided.

Methodology

Sample preparation	HEK293 cells were transfected with the vaccine, 48 hrs after they were stained with a live dead cell discriminator and a commercial polyclonal anti-SARS-CoV antibody (PA1-41165, Thermo Fisher Scientific)
Instrument	LSRForterssa (BD Biosciences)
Software	FACSDiva software (BD Biosciences), data were analyzed using FlowJo Version 10 (FlowJo LLC).

Cell population abundance	The positive population was identified using similarly stained but vaccine un-transfected cells to set the positivity cut-off.
Gating strategy	Cells were gated first on FSC/SSC for cells, then on FSA/FSH for single cells, then FSC/Aqua for live cells, and then FITC/FSC for SARS-CoV spike protein cells.The gating strategy is outlined in the supplementary material (Figure S1b) and uses a single cell gate followed by a live dead cell gate and finally a simple positive gate.

☒ Tick this box to confirm that a figure exemplifying the gating strategy is provided in the Supplementary Information.
