# Supplementary material for: Self-amplifying RNA SARS-CoV-2 lipid nanoparticle vaccine candidate induces high neutralizing antibody titers in mice
Source: Nat Commun. 2020 Jul 9;11:3523. doi: 10.1038/s41467-020-17409-9 (PMC7347890; doi:10.1038/s41467-020-17409-9)
Supplement: Supplementary file 2 — Peer Review File [file 41467_2020_17409_MOESM2_ESM.pdf]

Editorial Note: This manuscript has been previously reviewed at another journal that is not operating a transparent peer review scheme. This document only contains reviewer comments and rebuttal letters for versions considered at Nature Communications .

Reviewers' Comments:
Reviewer #1: Remarks to the Author:
The authors have adequately addressed this reviewer's comments

REVIEWERS' COMMENTS:
Reviewer #1 (Remarks to the Author):

The authors have adequately addressed this reviewer's comments.

Response: Thank you for your thorough review.
